# Lymphoepithelial Cyst “En Crypto”: A Case Report of a Rare Localization in the Superior Pole of the Palatine Tonsil

**DOI:** 10.3390/reports9010011

**Published:** 2025-12-29

**Authors:** Constantinos Papadopoulos, Konstantina Dinaki, Anastasia Sarafidou, Maria Peponi, Rafail Ioannidis

**Affiliations:** 1 Department of ENT Surgery, General Hospital of Drama, 66100 Drama, Greece; constantine.mbg@gmail.com (C.P.); peponmari1@gmail.com (M.P.); 2 Department of ENT Surgery, General Hospital of Mytilene Vostaneio, 81132 Lesvos, Greece; konnidin@windowslive.com; 3 Department of ENT Surgery, St. Luke’s Hospital, 55236 Thessaloniki, Greece; tesisaraf@gmail.com; 4 Department of Anesthesiology and Pain Medicine, General Hospital of Drama, 66100 Drama, Greece

**Keywords:** lymphoepithelial cysts, en crypto, superior tonsillar pole, tonsillar manipulation, case report

## Abstract

**Background and Clinical Significance:** Lymphoepithelial cysts are uncommon benign lesions of the head and neck, rarely encountered within the oral cavity and exceedingly infrequent in the palatine tonsils. Their nonspecific clinical presentation and ability to mimic more common benign entities often render diagnosis challenging. **Case Presentation**: We report the case of a 68-year-old woman with a four-year history of persistent foreign-body sensation in the oropharynx despite multiple normal otolaryngologic examinations. Flexible nasoendoscopy was non-diagnostic, as the lesion was deeply concealed within the superior tonsillar pole between the palatine pillars. Targeted tonsillar manipulation ultimately revealed a small pedunculated mass and contrast-enhanced computed tomography demonstrated a well-circumscribed, non-enhancing cystic lesion confined to the tonsillar parenchyma. Surgical excision under general anesthesia confirmed an oral lymphoepithelial cyst on histopathologic analysis. The patient remained asymptomatic with no recurrence at four months. **Conclusions**: This case underscores the rarity of tonsillar lymphoepithelial cysts, highlights the diagnostic limitations of endoscopic evaluation for cryptic superior-pole lesions and emphasizes the importance of meticulous dynamic oropharyngeal examination. Complete surgical excision is both definitive and curative, with an excellent prognosis.

## 1. Introduction and Clinical Significance

Lymphoepithelial cysts (LECs) are uncommon benign lesions characterized by a stratified squamous epithelial lining embedded within a dense lymphoid stroma containing germinal centers [1]. Although traditionally associated with the major salivary glands—particularly the parotid—LECs may occur throughout the head and neck region, including the submandibular area, cervical lymph nodes and, more rarely, the oral cavity [1,2]. Their etiopathogenesis remains incompletely defined, with proposed mechanisms including developmental epithelial entrapment within lymphoid aggregates, obstruction of minor salivary ducts, or epithelial proliferation triggered by chronic inflammatory or immune-mediated stimuli [2]. Clinically, LECs manifest as slow-growing, painless, well-demarcated nodules that can mimic a wide spectrum of benign pathologies, such as mucoceles, dermoid cysts, and minor salivary gland tumors, thus posing a diagnostic challenge [1,2]. Imaging modalities such as ultrasound and computed tomography (CT) may delineate their cystic architecture, but a definitive diagnosis requires histopathologic confirmation showing the characteristic epithelial–lymphoid interface [2]. Fine-needle aspiration may yield supportive but non-specific findings. Intraoral LECs represent a particularly rare subset, accounting for significantly fewer than 1% of all LECs in the head and neck [1,3]. The floor of the mouth and the ventral tongue surface are the most commonly affected sites; tonsillar or oropharyngeal presentations are exceedingly uncommon and have been documented primarily in isolated case reports [3,4,5,6,7]. Recent multicenter and population-based analyses have further emphasized their rarity but also highlighted consistent clinicopathologic features across large cohorts [7]. LECs of the oral cavity and oropharynx have been recognized not only as sporadic benign lesions but also in association with immunocompromised states, most notably human immunodeficiency virus (HIV) infection, where they may occur as part of diffuse lymphoid hyperplasia. Given their ability to clinically resemble other benign oral lesions, heightened awareness among clinicians is essential to prevent misdiagnosis and unnecessary interventions. Surgical excision is considered curative, with recurrence reported as exceedingly rare [1,2,7].

## 2. Case Presentation

**Figure 1:**
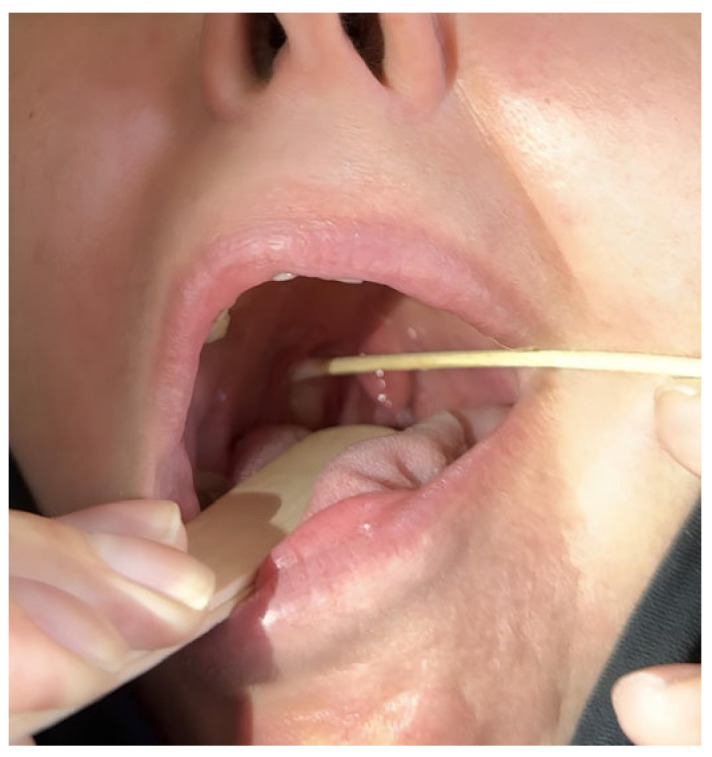
The lesion becomes evident only after dislocation of the right palatine tonsil, arising from its upper pole and remaining hidden in the tonsillar crypts, completely invisible on routine oropharyngeal inspection.

**Figure 2:**
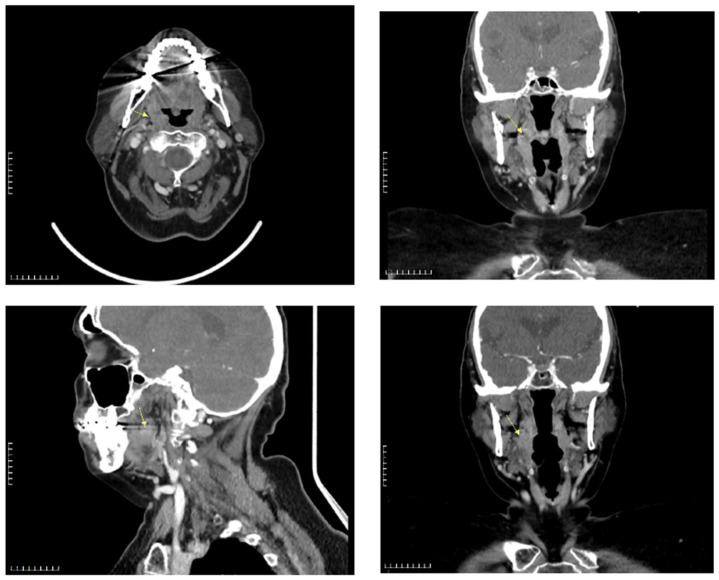
Preoperative CT images.

**Figure 3:**
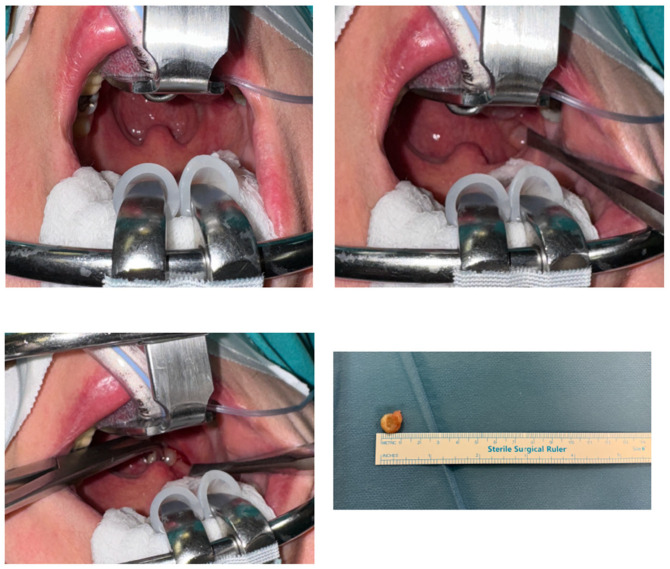
Intraoperative images and histopathology specimen.

**Figure 4:**
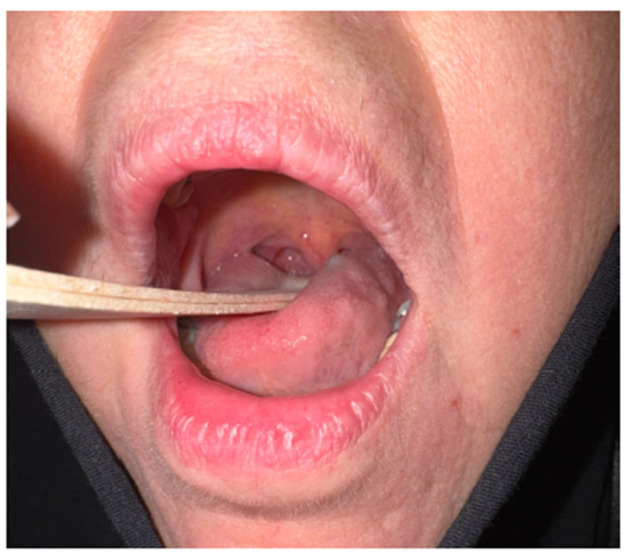
Postoperative oropharyngeal appearance at 4 months.

A 68-year-old female patient presented with a persistent foreign-body sensation in the oropharynx for approximately four years, without associated pain or dysphagia. Her medical history was notable for dyslipidemia, an episode of pulmonary thromboembolism and gastroesophageal reflux disease managed pharmacologically. Clinical examination by three independent otolaryngologists in the neutral position was unremarkable, with no palpable cervical lymphadenopathy. Laboratory testing showed no evidence of systemic inflammation. Over the four years preceding diagnosis, the patient experienced recurrent, self-limited episodes of a persistent foreign-body sensation in the pharynx. During the first year, two episodes lasting 7–10 days occurred, with the second approximately three months after the first and without clinical or laboratory evidence of inflammation. In the second year, three similar episodes were reported, beginning eight months after the last episode of the first year and followed by two further episodes at intervals of two and three months, respectively, all without inflammatory findings. A single episode occurred in the third year, seven months after the final episode of the second year, again in the absence of objective abnormalities. During the fourth year, one further episode of comparable duration occurred, during which the lesion was identified on clinical examination (Table 1). Until that time, all episodes were characterized by the absence of objective clinical or laboratory findings. Given the chronicity of symptoms, a detailed examination of the stomatopharynx was undertaken. Flexible nasoendoscopy did not reveal any lesion or pathological findings, as the cyst was deeply concealed within the upper tonsillar pole, situated between the anterior and posterior palatine pillars. Gentle medial displacement of the right palatine tonsil revealed a small, pedunculated, elastic, non-tender mass arising from and concealed within its superior pole (“en crypto” Greek etymology) (Figure 1). Contrast-enhanced CT demonstrated a well-circumscribed, non-enhancing cystic structure confined to the tonsillar parenchyma (Figure 2). Preoperative virological screening revealed no abnormalities, including negative HIV testing. The lesion was surgically excised under general, opioid-free anesthesia with endotracheal intubation (Figure 3). Histopathologic evaluation confirmed the diagnosis of an oral lymphoepithelial cyst, with no evidence of malignancy. At 4-month follow-up, the patient remained asymptomatic with no signs of recurrence (Figure 4).

**Table 1:** Chronological overview of symptom recurrence, diagnostic evaluation, and management over a four-year period prior to diagnosis.

Timeframe	Clinical Events	Duration of Symptoms	Clinical/Laboratory Findings
Year 1	Episode 1	7–10 days	No clinical or laboratory evidence of inflammation
	Episode 2 (≈3 months later)	7–10 days	No inflammatory findings
Year 2	Episode 3 (≈8 months after last Year 1 episode)	7–10 days	No clinical or laboratory abnormalities
	Episode 4 (≈2 months later)	7–10 days	No inflammatory findings
	Episode 5 (≈3 months later)	7–10 days	No inflammatory findings
Year 3	Episode 6 (≈7 months after last Year 2 episode)	7–10 days	No objective abnormalities
Year 4	Episode 7	7–10 days	Lesion identified during clinical examination
Diagnostic workup	Flexible nasoendoscopy	—	Non-diagnostic
	Targeted tonsillar manipulation	—	Pedunculated tonsillar mass revealed
	Contrast-enhanced CT	—	Well-circumscribed, non-enhancing cyst
Treatment	Surgical excision (opioid-free anesthesia)	—	Complete excision
Follow-up (4 months)	Clinical evaluation	—	Asymptomatic, no recurrence

## 3. Discussion

LECs of the oral cavity are uncommon benign lesions that most frequently arise in areas rich in lymphoid tissue, such as the floor of the mouth, ventral tongue and soft palate. Their occurrence within the palatine tonsil is particularly rare, accounting for only a small minority of reported cases in large clinicopathologic series. In the comprehensive review by Cunha et al., which analyzed 132 oral LECs, none originated from the palatine tonsils, underscoring the exceptional nature of the tonsillar location [7]. Similarly, the 26-case series by Sykara et al. reported no tonsillar involvement, with the floor of mouth and tongue predominating as primary sites [1]. Thus, a LEC arising from the superior pole of the palatine tonsil, particularly one concealed deep within the tonsillar crypts—as documented in our patient—represents a distinctly uncommon anatomical presentation.

The pathogenesis of oral LECs remains incompletely elucidated. The most widely accepted theory posits that they result from obstruction and cystic dilation of epithelial inclusions within lymphoid aggregates, possibly due to mucosal entrapment or crypt occlusion [2]. Palatine tonsils, which contain numerous mucosal invaginations (crypts), offer an anatomic substrate supportive of this mechanism. Case reports describing tonsillar LECs—including those by Moon et al. [4], Bingöl et al. [5], Castro et al. [6] and Shah et al. [3]—suggest that epithelial debris trapped within crypts may undergo cystic transformation, consistent with the “cryptogenic” hypothesis. However, unlike previously reported cases, the lesion in our patient was located entirely within the upper tonsillar pole and fully concealed (“en crypto”), making it clinically occult in standard oropharyngeal examination. This supports the possibility that LECs in this location may be underrecognized due to their deep cryptic origin.

Oral LECs are usually asymptomatic and discovered incidentally, although patients may report local discomfort or a foreign-body sensation [1,2]. Tonsillar LECs, while rare, similarly present with nonspecific symptoms including globus sensation, mild dysphagia, or local irritation [3,4,5,6]. In our case, the patient’s isolated, longstanding foreign-body sensation—without pain, dysphagia, or inflammatory findings—aligns with the benign clinical profile noted in prior literature.

A notable challenge in this case was the failure of multiple ear, nose, throat (ENT) examinations to identify the lesion, initially leading to diagnostic uncertainty. The cryptic position of the mass rendered it invisible in standard oropharyngeal inspection until targeted palpation displaced the right tonsil medially, revealing the pedunculated cyst. Such deep-crypt lesions emphasize the importance of thorough oropharyngeal evaluation, especially when symptoms persist in the absence of objective findings.

Flexible nasoendoscopy was not as diagnostically informative as anticipated in this case. This contrasts with previous recommendations suggesting that endoscopic evaluation enhances the detection of tonsillar anomalies, particularly when lesions are situated beyond the direct line of sight [4,5].

Cross-sectional imaging plays a supplementary role in diagnosis. Contrast-enhanced CT in this case demonstrated a well-circumscribed, non-enhancing cystic lesion, consistent with findings from earlier reports [4,5,6]. Radiologic evaluation is especially useful in distinguishing LECs from alternative cystic or solid lesions of the tonsil, such as mucus retention cysts, epidermoid cysts, or neoplastic processes. On contrast enhanced CT, LECs in the tonsillar region typically present as well defined, non-enhancing, low attenuation cystic lesions that are superficially located within or adjacent to the tonsillar tissue and lack aggressive features such as surrounding soft tissue invasion or regional lymphadenopathy [4,8]. The contents generally demonstrate homogeneous fluid attenuation consistent with simple cystic material. In contrast, intratonsillar epidermoid (epidermal inclusion) cysts—though rare—can also appear as well circumscribed low-density lesions on CT; however, they may show subtle heterogeneity in attenuation due to keratinous debris within the cyst cavity, a feature that is less typical for simple lymphoepithelial cysts [3]. Mucus retention cysts, on the other hand, are usually small and closely linked to obstructed minor salivary ducts without distinctive CT features beyond homogeneous fluid attenuation, while cystic neoplastic lesions tend to exhibit irregular margins, internal septations, or contrast enhancement [8,9]. Careful evaluation of lesion location, attenuation characteristics and enhancement behavior on CT can thus aid in narrowing the differential diagnosis of benign cystic lesions of the tonsil.

Intraoral LECs are most frequently described as small, smooth nodules of the floor of the mouth or lateral tongue and are rarely noted in the tonsillar region, where reports are limited to a small number of case studies [3,4]. While the literature does not specifically quantify the frequency of pedunculated versus sessile presentations, the typical descriptions of oral LECs in the floor of the mouth emphasize a sessile, submucosal nodule morphology, consistent with their origin from superficial lymphoid tissue [1]. In contrast, tonsillar LECs have occasionally been reported as discrete, well-circumscribed lesions arising from the tonsillar crypts, which in our case may manifest with a pedunculated appearance due to the anatomy and mobility of the tonsillar tissue [3,4]. This distinction in clinical presentation enriches the anatomical context of the lesion and highlights that tonsillar LECs may present with varied morphologies depending on their site of origin within the lymphoid architecture.

The relationship between LECs and HIV infection has been well documented in the literature, particularly in the context of diffuse hyperplastic lymphoid lesions of the oral and oropharyngeal mucosa [10]. In individuals with HIV, chronic antigenic stimulation and immune dysregulation are thought to drive lymphoid proliferation, which in turn may predispose to the formation of benign cystic structures within lymphoid-rich tissues such as the tonsils and floor of the mouth. These HIV-associated lymphoid lesions can present clinically as multiple, bilateral, or unusually large cystic masses, a pattern that differs from the typically solitary and localized presentation seen in immunocompetent patients. The potential association with HIV emphasizes the importance of a thorough clinical and immunological evaluation when LECs occur in atypical distributions, multifocal patterns, or in patients with risk factors for immune compromise. Although our patient did not demonstrate clinical or laboratory evidence of HIV infection, awareness of this association remains relevant for comprehensive differential diagnosis and patient care [11].

Histopathologic examination remains the gold standard for diagnosis. Classic microscopic features include a cystic cavity lined by keratinizing stratified squamous epithelium surrounded by dense lymphoid tissue with germinal centers, as detailed extensively in the clinicopathologic studies by Sykara et al. [1] and Cunha et al. [7]. The histologic findings in our case were fully concordant with these criteria and, importantly, demonstrated no dysplasia or malignant transformation.

Surgical excision is the treatment of choice for oral LECs and is curative in nearly all cases [1,2,7]. Excision of tonsillar LECs can be performed via local or general anesthesia depending on the depth and accessibility of the lesion. Our decision for removal under opioid-free general anesthesia with endotracheal intubation ensured adequate exposure of the cryptic superior pole.

Recurrence after complete excision has not been reported in the literature, regardless of location [1,2,7]. Consistent with these observations, our patient remains asymptomatic with no evidence of recurrence at the 4-month follow-up.

This case contributes to the limited literature on palatine tonsillar LECs by highlighting several clinically relevant aspects: (1) A rare anatomical location (superior tonsillar pole) that has been scarcely reported. (2) A cryptic, completely hidden lesion, explaining the initially negative ENT evaluations. (3) Isolated foreign-body sensation without other symptoms, consistent with benign behavior. (4) Diagnostic value of targeted tonsillar manipulation and endoscopic assessment. (5) Excellent postoperative outcome, confirming the benign nature and curative potential of surgical excision.

## 4. Conclusions

This report presents a rare case of an oral lymphoepithelial cyst arising from the superior pole of the palatine tonsil, clinically visible only after tonsillar displacement. The case highlights the critical importance of dynamic and meticulous oropharyngeal examination in patients with persistent, low-grade symptoms despite unremarkable routine inspection. Definitive diagnosis relies on histopathologic evaluation and complete surgical excision remains both diagnostic and curative.

## Data Availability

The original contributions presented in this study are included in the article. Further inquiries can be directed to the corresponding author.

## Abbreviations

The following abbreviations are used in this manuscript:

LECs	Lymphoepithelial cysts
CT	Computed tomography
HIV	Human immunodeficiency virus
ENT	Ear, nose, throat
